# Gain and loss of function mutations in biological chemical reaction networks: a mathematical model with application to colorectal cancer cells

**DOI:** 10.1007/s00285-021-01607-0

**Published:** 2021-05-04

**Authors:** Sara Sommariva, Giacomo Caviglia, Michele Piana

**Affiliations:** 1 Dipartimento di Matematica, Universitá di Genova, Via Dodecaneso, 35 16146 Genoa, Italy; 2 CNR - SPIN GENOVA, Via Dodecaneso, 35 16146 Genoa, Italy

**Keywords:** Reaction kinetics, Synthetic cell biology, Loss of function mutations, Gain of function mutations, Colorectal cancer cells, G1-S transition point, 92C42 (System biology,networks), 92-10 (Mathematical modeling or simulation for problems pertaining to biology), 37M05 (Simulation of dynamical systems)

## Abstract

This paper studies a system of Ordinary Differential Equations modeling a chemical reaction network and derives from it a simulation tool mimicking Loss of Function and Gain of Function mutations found in cancer cells. More specifically, from a theoretical perspective, our approach focuses on the determination of moiety conservation laws for the system and their relation with the corresponding stoichiometric surfaces. Then we show that Loss of Function mutations can be implemented in the model via modification of the initial conditions in the system, while Gain of Function mutations can be implemented by eliminating specific reactions. Finally, the model is utilized to examine in detail the G1-S phase of a colorectal cancer cell.

## Introduction

Signaling networks are Chemical Reaction Networks (CRNs) consisting of an interconnected set of pathways, modeling the flow of chemical reactions initiated by information sensed from the environment through families of receptor ligands (Jordan et al. 2000; Sever and Brugge 2015; Tyson and Novak 2008). The reactions in the network follow the process of information transfer inside cytosol down to the description of the activity of a few related transcription factors (Karin and Smeal 1992; Kohn et al. 2006). Similarly to what happens for many networks of biological interest, a signaling network may comprise hundreds of reacting chemical species and reactions.

Any CRN can be mapped into a system of ordinary differential equations (ODEs) by following standard procedures (Feinberg 1987; Yu and Craciun 2018; Chellaboina et al. 2009) thus allowing in particular for simulations of the kinetics of the signaling process in the biologic case (see, e.g., Anderson et al. 2019; Roy and Finley 2017). The state variables of such a system of ODEs are rapresented by the concentration of the reacting chemical species. Fixed an initial state, the solution of the system thus simulates the time-varying transient behavior of the species concentrations which evolve until in the long term the network usually reaches an (asymptotic) stable state, that is also an equilibrium state of the system (Ingalls 2013). The number of allowable steady states and their stability properties depend on the system characteristics and have been the subject of intensive studies since 1970s (Érdi and Tóth 1989), also in view of practical implications such as, e.g., analysis of activation of the ERK cascade (Conradi and Flockerzi 2012; Conradi and Mincheva 2014). For example, uniqueness of the steady states has been investigated through indices based on the network topology, such as the deficiency index introduced by Feinberg (1987), or through analytical approaches based on the analysis of the Jacobian of the system of ODEs for mass-action networks (see e.g. the review paper by Yu and Craciun (2018) and the cited references). Adopting an alternative viewpoint, Conradi and co-workers analyzed the problem of characterizing multistationarity (Conradi and Flockerzi 2012; Conradi and Mincheva 2014), a necessary prerequisite for bistability which in turn enables properties like sharp response thresholds and hysteresis for phosphorylation networks (Conradi and Mincheva 2014).

In the present work we start from a CRN modeling physiological (i.e. healthy) states and we show how it can be formally modified to simulate individual pathological conditions associated to particular classes of mutations. We compute and analyze the asymptotic stable states of both the systems, mimicking either a healthy or a pathological condition, in order to quantify the changes in the species concentrations induced by mutations. Since most cancer diseases result from an accumulation of a set of mutations (Stratton et al. 2009; Levine et al. 2019), the modeling of a family of mutations and the numerical solution of the related system of ODEs represents a convenient computational tool for the simulation of the mechanisms giving rise to a tumor. Further, this kind of models can typically be tuned in order to mimic the effects of targeted therapies (Facchetti et al. 2012; Levine 2019) and drug resistance mechanisms (Eduati et al. 2017).

More in details, we first realize an analysis of the system of ODEs associated to a CRN, characterized by a level of generality sufficient to provide an efficient simulation tool. From a formal viewpoint, we apply mathematical methods devised for the investigation of deterministic homogeneous chemical reaction systems based on mass-action kinetics (Chellaboina et al. 2009). The use of mass-action over more complex kinetics, such as e.g. Michaelis–Menten or Hill kinetics, is motivated by the fact that, in our signal transduction network, the substrates of enzymatic activity are proteins, whose concentration is comparable to that of catalysing enzymes (Ingalls 2013).

A crucial role in our approach is played by moiety conservation laws (CLs), which are essential in the determination and interpretation of results (De Martino et al. 2014; Shinar et al. 2009). They may be regarded as an analogue of the conservation relations extensively used in the papers of Conradi and co-workers, in that two multistationary states must satisfy the same conservation conditions. However, our approach to steady states is essentially numerical and is discussed by relying on a geometric classification of equilibrium states in terms of stoichiometric surfaces, together with their stability properties.

From a more operating viewpoint, we define projection operators, which map the system of ODE for physiological conditions into the mutated system which models Loss of Function (LoF) and Gain of Function (GoF) mutations (Griffiths et al. 2005; Li et al. 2019). The model is built and made operative in such a way that it can be modified almost straightforwardly by addition or elimination of chemical reactions or chemical species, change in the values of the rate constants in the formulation of mass-action laws, or change of the initial conditions. As a consequence, the parameter values that have been originally considered as fixed can be customized to fit the tumor data of a specific patient.

As an application, we examine in detail the kinetics of a recently proposed network which simulates how colorectal cancer (CRC) cells process information from external growth factors and the related answer (Tortolina et al. 2015). The network is focused on the G1-S transition point. In this transition a newborn cell in the G1 phase of its cycle must pass the control of this checkpoint before starting the S phase of synthesis of new DNA (Tyson and Novak 2008); indeed, this is the first necessary step towards proliferation.

The structure of the paper is as follows. Section 2 revisits classical results on CRNs modeling required to understand our model for LoF and GoF mutations, and defines the notations used throughout the paper. Section 3 contains the mathematical model describing LoF and GoF mutation processes. Section 4 is devoted to the application of the model to CRC cells. Our conclusions are offered in Sect. 5.

## Chemical reaction networks for cell signaling

We consider a CRN consisting of *r* reactions, denoted as $R_j$, $j=1, \dots , r$, that involve *n* well-mixed reacting species, denoted as $A_i$, $i=1, \dots , n$. The network is modeled as a dynamical system with state vector $\mathbf {x} =(x_1, \dots , x_n)^T \in {\mathbb {R}}^n_+$, where the upper *T* denotes transposition, ${\mathbb {R}}_+$ is the set of non-negative real numbers, and the generic component $x_i$ is the molar concentration (nM) of the species $A_i$. According to this chemical interpretation, $\mathbf {x}$ is also called concentration vector (Yu and Craciun 2018). We assume that the law of mass action holds: when two or more reactants are involved in a reaction, the reaction rate is proportional to the product of their concentrations. The resulting polynomial system of ODEs for the state variables is written as1where the superposed dot denotes the time derivative; $\mathbf {k}=\left( k_1, \dots , k_r \right) ^T \in {\mathbb {R}}^r_+$ stands for the set of rate constants; $\mathbf {S}$ is the ${\mathbb {R}}^{n \times r}$ constant stoichiometric matrix, $\mathbf {v}(\mathbf {x},\mathbf {k}) \in {\mathbb {R}}^r_{+}$ is the vector of reaction fluxes. Here, system (1) accounts for internal reaction and boundary fluxes (Kschischo 2010; Schilling et al. 2000). Thus in general equation (1) describes an *open* network, where inflow processes are accounted for by enlarging the system of ODEs through additional constant species and there are non-zero outflow/degradation rates for some of the species (Feinberg 1987; Yu and Craciun 2018). The matrix element $S_{ij}$ is the net number of molecules of the species $A_i$ that are produced whenever the reaction $R_j$ occurs. Thus the columns of ${\mathbf {S}}$ have been referred to as reaction vectors.

We are now interested in investigating the general properties of the solutions of the system (1), and, in particular, in computing the corresponding asymptotically stable states. Our study is based on the analysis of the conservation laws and the stoichiometric compatibility classes of the system revised in the next two subsections.

### Conservation laws and elemental species

#### Definition 1

Let $\mathbf {x}(t)$ be a solution of the system of ODEs (1). A constant vector $\varvec{\gamma } \in {\mathbb {N}}^n \setminus \left\{ \mathbf {0} \right\}$ is said to be a *semi-positive conservation vector* if there exists $c \in {\mathbb {R}}_+$ such that2Moreover, the relation (2) is called a *semi–positive conservation law*, or equivalently *moiety conservation law*.

Since concentrations are expressed in nM, the biochemical interpretation of the conservation law is that the total number of molecules involved in the species combination ${\varvec{\gamma }}^T \mathbf {x}$ remains constant during the evolution in time of the network (Shinar et al. 2009). The constant total amount of available molecules is fixed by ${\varvec{\gamma }}$ and the initial state $\mathbf {x}_0$ through $c = {\varvec{\gamma }}^T \mathbf {x}_0$. Moreover, the concentrations of the species involved in the conservation law are bounded from above by the constant *c* (De Martino et al. 2014; Haraldsdóttir and Fleming 2016; Shinar et al. 2009; Schuster and Höfer 1991). The following proposition relates the conservation vectors to the properties of the stoichiometric matrix.

#### Proposition 1

If ${\varvec{\gamma }}\in \ker (\mathbf {S}^T) \cap {\mathbb {N}}^n \setminus \left\{ \mathbf {0} \right\}$, then ${\varvec{\gamma }}$ is a conservation vector.

#### Proof

The thesis follows by observing that for any solution $\mathbf {x}(t)$ of the system of ODEs (1)

$$\begin{aligned} \frac{d}{dt} ({\varvec{\gamma }}^T \mathbf {x}) = {\varvec{\gamma }}^T \dot{\mathbf {x}} = {\varvec{\gamma }}^T \mathbf {S} \mathbf {v}(\mathbf {x}, \mathbf {k}) = 0, \end{aligned}$$

where the last term is equal to zero as ${\varvec{\gamma }}\in \ker (\mathbf {S}^T)$. $\square$

We concentrate on conservation vectors ${\varvec{\gamma }}\in \ker (\mathbf {S}^T)$. Proposition 1 implies that the set of all possible semi–positive conservation vectors defines a convex cone whose independent generators can be computed e.g. through the algorithm proposed by Schuster and Höfer (1991). In the following we will denote with $\left\{ {\varvec{\gamma }}_1, \dots , {\varvec{\gamma }}_p \right\}$ a set of such generators and define the matrix $\mathbf {N} \in {\mathbb {R}}^{p \times n}$ as

3 $$\begin{aligned} \mathbf {N} = \begin{bmatrix} {\varvec{\gamma }}_1^T \\ \vdots \\ {\varvec{\gamma }}_{p}^T \end{bmatrix} . \end{aligned}$$

Henceforth we will assume $p = n - \mathrm{rank}(\mathbf {S})$ and thus the set $\left\{ {\varvec{\gamma }}_1, \dots , {\varvec{\gamma }}_p \right\}$ defines a basis for $\ker (\mathbf {S}^T)$.

Denote by $\mathbf {x}_0$ the initial point of a trajectory. Since $\mathbf {N} \mathbf {S} = 0$, it follows that

4 $$\begin{aligned} \mathbf {N} \mathbf {x}(t) =\mathbf {N} \mathbf {x}_0=: \mathbf {c} \end{aligned}$$

is the constant vector in ${\mathbb {R}}^p$ whose components are the constants involved in the CLs. Thus the matrix $\mathbf {N}$ determines *p* linear, independent CLs; accordingly, the representative point $\mathbf {x}(t)$ is constrained to move on the affine subspace of ${\mathbb {R}}^n$ which is determined by Eq. (4), and hence is identified by $\mathbf {N}$ and the initial data. Moreover, the linear affine subspace is the intersection of *p* hyperplanes.

We conclude with a few remarks that will play a fundamental role in the analysis of the solutions of system (1). In general, there are chemical species that are not involved explicitly in the expressions of the CLs while the remaining species may belong to more than one CL.

#### Definition 2

We say that a CRN is *elemented* if: (i) it admits a set of generators $\left\{ \varvec{\gamma }_1, \dots , \varvec{\gamma }_p \right\} \subset {\mathbb {N}}^n$ such that the matrix $\mathbf {N}$ contains at least one minor equal to the identity matrix of order *p*, say $\mathbf {I}_p$; (ii) each chemical species is involved in at least one CL, i.e. for each $i=1, \dots , n$ there exists $k \in \{1, \dots , p\}$ such that $\gamma _{ki} \ne 0$. If only condition (i) is fulfilled, the CRN is *weakly elemented*.

Borrowing the terminology of Shinar et al. (2009), the species associated with the minor equal to the identity matrix will be called *elemental* or *basic species*.

#### Remark 1

Condition (ii) in Definition 2 is equivalent to the requirement that the CRN is *conservative*, i.e. that there exists ${\mathbf {m}} \in {\mathbb {R}}^n_+$, s.t. $m_i > 0$ for all $i = 1, \dots , n$, and ${\mathbf {S}}^T {\mathbf {m}} = 0$ (Érdi and Tóth 1989; Schuster and Höfer 1991).

#### Remark 2

Definition 2 implies that each elemental species belongs to one, and only one conservation law. The idea is that elemental species consist of proteins in free form, which bind to other species involved in the same conservation law, in order to form the derived compounds or secondary species.

In general the set of elemental species of a network is not unique as it depends on the choice of the basis of the conservation vectors and, fixed a basis, multiple minors equal to the identity matrix may exist.

#### Remark 3

Given a weakly elemented CRN, up to a change in the order of the components of $\mathbf {x}$, the matrix $\mathbf {N}$ may be decomposed as

5 $$\begin{aligned} \mathbf {N} = \left[ \mathbf {I}_p, \mathbf {N}_2 \right] \end{aligned}$$

with $\mathbf {N}_2 \in {\mathbb {R}}^{p \times (n-p)}$. Similarly, the concentration vector $\mathbf {x}$ may be decomposed as

6 $$\begin{aligned} \mathbf {x} = \left( \begin{array}{c} \mathbf {x}_1 \\ \mathbf {x}_2 \end{array} \right) \end{aligned}$$

with $\mathbf {x}_1 \in {\mathbb {R}}^p$, and $\mathbf {x}_2 \in {\mathbb {R}}^{n-p}$. In particular, $\mathbf {x}_1 \in {\mathbb {R}}^p$ is formed by the elemental variables. Thus Eq. (4) may be rewritten in the equivalent form

7 $$\begin{aligned} \mathbf {x}_1 = \mathbf {c} - \mathbf {N}_2 \, \mathbf {x}_2 \end{aligned}$$

Henceforth we assume the CRN to be weakly elemented, with elemental variables $x_1 \ldots x_p$, so that the matrix $\mathbf {N}$ is described by the block decomposition (5).

According to (7), the set of *p* conservation equations is solved straightforwardly with respect to the elemental variables, thus yielding a parametric description of the affine space defined in (4). Furthermore, substitution of the expression (7) of $\mathbf {x}_1$ into the system (1) provides a reduced formulation of the original system of ODEs.

#### Definition 3

Consider a weakly elemented CRN. A concentration vector $\mathbf {x} \in {\mathbb {R}}^n$ is an *ideal state* for the network iff only the elemental species have non-zero concentration, i.e., referring to Eq. (6), $\mathbf {x}_2 = 0$.

### Stoichiometric compatibility classes

Given the initial condition $\mathbf {x}(0)=\mathbf {x}_0$, consider the corresponding solution $\mathbf {x}(t)$ of the system (1), defined at least in the time domain [0, *T*]. Integration in time of both sides of (1) leads to

8 $$\begin{aligned} \mathbf {x}(t) - \mathbf {x}_0 = \mathbf {S} \, \int _0^t \mathbf {v}(\mathbf {x}(\tau ), \mathbf {k}) \, d\tau , \quad t \in [0,T] . \end{aligned}$$

This shows that $\mathbf {x}(t)-\mathbf {x}_0$ is a linear combination of the reaction vectors, with time-dependent coefficients. Therefore $\mathbf {x}(t)-\mathbf {x}_0$ belongs to a vector space of dimension equal to rank $(\mathbf {S})$ defined by the image of the stoichiometric matrix (Feinberg 1987, 1995; Yu and Craciun 2018). Accordingly, we provide the following definition of *stoichiometric compatibility class* (SCC).

#### Definition 4

Given a value $\mathbf {x} \in {\mathbb {R}}^n_+$ of the state variable for system (1), we define a *stoichiometric compatibility class* (SCC) of $\mathbf {x}$ the set

9 $$\begin{aligned} {\mathcal {S}}{\mathcal {C}}(\mathbf {x}) = \left( \mathbf {x} + \mathrm{span}(\mathbf {S}) \right) \cap {\mathbb {R}}^n_+ . \end{aligned}$$

#### Proposition 2

Let $\left\{ {\varvec{\gamma }}_1, \dots , {\varvec{\gamma }}_p \right\}$ be a set of generators of the the convex cone defined by the semi–positive conservation laws and let $\mathbf {N}$ be the matrix defined as in Eq. (5). If $p = n - \mathrm{rank}(\mathbf {S})$ then for all $\mathbf {x} \in {\mathbb {R}}^n_+$

10 $$\begin{aligned} {\mathcal {S}}{\mathcal {C}}(\mathbf {x})&= \left( \mathbf {x} + \ker (\mathbf {S}^T)^{\perp } \right) \cap {\mathbb {R}}^n_+ \end{aligned}$$

11 $$\begin{aligned}&= \left\{ \mathbf {y} \in {\mathbb {R}}^n_+\ \mathrm{s.t.}\ \mathbf {N}\mathbf {y} = \mathbf {N}\mathbf {x} \right\} . \end{aligned}$$

#### Proof

Equation (10) simply follows from Definition 4 by observing that span$(\mathbf {S}) = (\ker (\mathbf {S}^T))^{\perp }$. Equation (11) follows from the fact that, since $p = n - \mathrm{rank}(\mathbf {S})$, the set $\left\{ {\varvec{\gamma }}_1, \dots , {\varvec{\gamma }}_p \right\}$ is a basis for $\ker (\mathbf {S}^T)$. $\square$

In this work, we are interested in computing the asymptotically stable states of system (1), that shall allow us to characterize and quantitatively compare the long-term values of the species concentrations in the healthy and mutated network. However system (1) has in general multiple equilibrium points. Motivated by this consideration and exploiting the presence of conservation laws, we define a condition that guarantees uniqueness and global stability of the steady state once fixed a SCC.

#### Definition 5

A CRN is said to satisfy the global stability condition if for every stoichiometric compatibility class there exists a unique globally asymptotically stable state $\mathbf {x}_e$.

This means in particular that every trajectory with initial point on the given SCC tends asymptotically to the steady state $\mathbf {x}_e$, which is also an equilibrium point. Details about asymptotic stability properties of CRNs can be found, e.g., in Chellaboina et al. (2009), Feinberg (1987) and Yu and Craciun (2018).

## Mathematical model of loss and gain of function mutations

A mutation consists essentially in a permanent alteration in the nucleotide sequence of the genome of a cell. Mutations play a fundamental role in cancer evolution (Stratton et al. 2009; Weinstein et al. 2013). Here we are concerned with effects induced by mutations on species concentrations in the CRN. Specifically, we consider two particular classes of mutations, namely LoF and GoF mutations, that are commonly observed in cancer cells (Hochman et al. 2017; Lemieux et al. 2015; Levine 2019; Levine et al. 2019).

In details, we consider LoF mutations that result in reduction or abolishment of the function of a protein $A_i$, which is simulated by a restriction on the value of its concentration $x_i$. The degree to which the function is lost can vary; for null mutations the function is completely lost and the concentration of the related molecules is supposed to vanish; for leaky mutations some function is conserved and the value of concentration is appropriately reduced (Griffiths et al. 2005; Li et al. 2019). In this study, we deal with null LoF mutations by referring directly to the concentrations of the mutated molecular species, and by assuming that they are set equal to zero.

As for the GoF mutations, we assume they are responsible for an enhanced activity of a specific protein, so that its effects become stronger (Griffiths et al. 2005; Li et al. 2019). In our framework a GoF is implemented by excluding from our CRN the reactions involved in the deactivation of the considered protein; this is achieved, by setting to zero the corresponding reaction rates.

### Loss of function

Consider a CRN described by system (1) and consider a state $\mathbf {x}$ of the system. A LoF mutation of the elemental species $A_j$ results in a projection of the state $\mathbf {x}$ in a novel state where the concentrations of the $j-$th elemental species and of all its compounds are zero. Equivalently, also the total concentration $c_j$ available in the $j-$th conservation law is zero. This is modeled by applying the following operator to the state $\mathbf {x}$.

#### Definition 6

Consider a weakly elemented CRN and let $A_j$ be the $j-$th elemental species of the network. A LoF mutation of $A_j$ results in the operator ${\mathcal {P}}_{L_j} : {\mathbb {R}}^n \rightarrow {\mathbb {R}}^n$ that projects $\mathbf {x}$ into a novel state ${\mathcal {P}}_{L_j}(\mathbf {x}) = \left[ \tilde{\mathbf {x}}^T_1 , \tilde{\mathbf {x}}^T_2\right] ^T$ such that

$$\begin{aligned} {\tilde{x}}_{2,i} = {\left\{ \begin{array}{ll} 0 &{} \text {if } \gamma _{ji} \ne 0 \\ x_{2, i} &{} \text {otherwise} \end{array}\right. }, \quad i=p+1, \dots , n \end{aligned}$$

and

$$\begin{aligned} \tilde{\mathbf {x}}_1 = \left( \widetilde{\mathbf {c}} - \mathbf {N}_2 \tilde{\mathbf {x}}_2 \right) \end{aligned}$$

where $\widetilde{\mathbf {c}}$ is obtained by setting to 0 the $j-$th element of the vector $\mathbf {c} = \mathbf {N} \mathbf {x}$.

#### Remark 4

If $\mathbf {x}$ is an ideal state for the CRN then ${\mathcal {P}}_{Lj}(\mathbf {x})$ is obtained by setting to zero the concentration $x_j$ of the $j-$th elemental species.

#### Remark 5

${\mathcal {P}}_{L_j}$ transforms SCCs into SCCs. Indeed, if the concentration vectors $\mathbf {x}$ and $\mathbf {y}$ belong to the same SCC, i. e.

$$\begin{aligned} \mathbf {N} \mathbf {x} = \mathbf {N} \mathbf {y} = \mathbf {c} , \end{aligned}$$

then ${\mathcal {P}}_{L_j} (\mathbf {x})$ and ${\mathcal {P}}_{L_j}(\mathbf {y})$ still belong to the same SCC. More in details,

$$\begin{aligned} \mathbf {N} {\mathcal {P}}_{L_j}(\mathbf {x}) = \mathbf {N} {\mathcal {P}}_{L_j}(\mathbf {y}) = \widetilde{\mathbf {c}} , \end{aligned}$$

where ${\widetilde{c}}_i = c_i \, (1-\delta _{ij})$, $i = 1, \dots , p$, $\delta _{ij}$ being the Kronecker delta, i.e. $\widetilde{\mathbf {c}}$ is equal to $\mathbf {c}$ except for the $j-$th element that is zero. This result follows straightforwardly from Definition 6 by observing that

$$\begin{aligned} \mathbf {N} {\mathcal {P}}_{L_j}(\mathbf {x}) = \left[ \mathbf {I}_p, \mathbf {N}_2 \right] \left( \begin{array}{c} \tilde{\mathbf {x}}_1 \\ \tilde{\mathbf {x}}_2 \end{array} \right) = \tilde{\mathbf {x}}_1 + \mathbf {N}_2 \tilde{\mathbf {x}}_2 = \widetilde{\mathbf {c}} - \mathbf {N}_2 \tilde{\mathbf {x}}_2 + \mathbf {N}_2 \tilde{\mathbf {x}}_2 = \widetilde{\mathbf {c}} , \end{aligned}$$

and analogously for $\mathbf {N} {\mathcal {P}}_{L_j}(\mathbf {y})$. Therefore the following theorem holds.

#### Theorem 1

Consider a weakly elemented CRN described by the system of ODEs (1), and any states $\mathbf {x}$, $\mathbf {y}$ such that ${\mathcal {S}}{\mathcal {C}}(\mathbf {x}) = {\mathcal {S}}{\mathcal {C}}(\mathbf {y})$. Then ${\mathcal {S}}{\mathcal {C}}({\mathcal {P}}_{L_j}(\mathbf {x})) = {\mathcal {S}}{\mathcal {C}}({\mathcal {P}}_{L_j}(\mathbf {y}))$.

If in addition the CRN satisfies the global stability condition then the trajectories starting from ${\mathcal {P}}_{L_j}(\mathbf {x})$ and ${\mathcal {P}}_{L_j}(\mathbf {y})$ lead to the same globally asymptotically stable state.

#### Proof

To prove the first part of the theorem we observe that ${\mathcal {S}}{\mathcal {C}}(\mathbf {x}) = {\mathcal {S}}{\mathcal {C}}(\mathbf {y})$ implies, in particular, $\mathbf {y} \in {\mathcal {S}}{\mathcal {C}}(\mathbf {x})$ and thus $\mathbf {N} \mathbf {x} = \mathbf {N} \mathbf {y}$. From Remark 5, it follows $\mathbf {N} {\mathcal {P}}_{L_j}(\mathbf {x}) = \mathbf {N} {\mathcal {P}}_{L_j}(\mathbf {y})$, that is ${\mathcal {P}}_{L_j}(\mathbf {y}) \in {\mathcal {S}}{\mathcal {C}}({\mathcal {P}}_{L_j}(\mathbf {x}))$, and thus ${\mathcal {S}}{\mathcal {C}}({\mathcal {P}}_{L_j}(\mathbf {x})) = {\mathcal {S}}{\mathcal {C}}({\mathcal {P}}_{L_j}(\mathbf {y}))$.

The second part of the theorem follows from Definition 5, since for every SCC there exist a unique global asymptotically stable state, and ${\mathcal {P}}_{L_j}(\mathbf {x})$ and ${\mathcal {P}}_{L_j}(\mathbf {y})$ belong to the same SCC. $\square$

#### Remark 6

This holds in particular if $\mathbf {x}$ and $\mathbf {y}$ are replaced by the initial state $\mathbf {x}_0$ and the corresponding steady state $\mathbf {x}_e$, respectively. Thus the trajectories starting from ${\mathcal {P}}_{L_j}(\mathbf {x}_0)$ and ${\mathcal {P}}_{L_j}(\mathbf {x}_e)$ lead to the same mutated steady state.

### Gain of function

Consider a CRN described by system (1). In particular, let $\mathbf {S}$ be the stoichiometric matrix of the system. A mutation resulting in the GoF of a given protein is implemented by removing from the network the reactions involved in the deactivation of such a protein. From a mathematical viewpoint this can be achieved by setting to zero the values of the corresponding rate constants, or equivalently by setting to zero the corresponding columns of $\mathbf {S}$.

#### Definition 7

Consider a CRN and let $\mathbf {S}$ be the corresponding stoichiometric matrix. Given a set of reactions identified by the indices $H \subseteq \left\{ 1, \dots , r \right\}$ a GoF mutation results in the operator ${\mathcal {G}}_H : {\mathbb {R}}^{n \times r} \rightarrow {\mathbb {R}}^{n \times r}$ that projects $\mathbf {S}$ into a novel stoichiometric matrix $\widetilde{\mathbf {S}} = {\mathcal {G}}_H(\mathbf {S})$ such that

$$\begin{aligned} {\tilde{S}}_{i, h} = {\left\{ \begin{array}{ll} 0 &{} \text {if } h \in H \\ S_{i, h} &{} \text {otherwise} \end{array}\right. }, \quad i=1, \dots , n~, \end{aligned}$$

#### Remark 7

${\mathcal {G}}_H(\mathbf {S})$ defines a new CRN where the chemical reactions in *H* have been removed, while the set of chemical species, and thus the state space ${\mathbb {R}}^n$, are kept fixed.

#### Theorem 2

If the set of reactions $H \subseteq \left\{ 1, \dots , r \right\}$ is such that

12 $$\begin{aligned} \mathrm{rank} \left( {\mathcal {G}}_H(\mathbf {S}) \right) = \mathrm{rank} \left( \mathbf {S} \right) \end{aligned}$$

then

13 $$\begin{aligned} \ker \left( {\mathcal {G}}_H(\mathbf {S})^T \right) = \ker \left( \mathbf {S}^T \right) \end{aligned}$$

and thus in particular the stoichiometric matrices ${\mathcal {G}}_H(\mathbf {S})$ and $\mathbf {S}$ define the same SCCs.

#### Proof

Using standard results from linear algebra, from Definition 7 it follows that $\ker \left( \mathbf {S}^T \right) \subseteq \ker \left( {\mathcal {G}}_H(\mathbf {S})^T \right) .$ Indeed, if ${\varvec{\gamma }}\in \ker \left( \mathbf {S}^T \right)$ then

$$\begin{aligned} \mathbf {0} = {\varvec{\gamma }}^T \mathbf {S} = \left( {\varvec{\gamma }}^T \mathbf {S}_1, \dots , {\varvec{\gamma }}^T \mathbf {S}_r \right) \end{aligned}$$

where $\mathbf {S}_j$, $j=1, \dots , r$, denotes the $j-$th column of $\mathbf {S}$. In particular ${\varvec{\gamma }}^T \mathbf {S}_j = 0$ for all $j = 1, \dots , r$, $j \not \in H$, that is ${\varvec{\gamma }}\in \ker \left( {\mathcal {G}}_H(\mathbf {S})^T \right)$.

Additionally, Eq. (12) implies that $\ker \left( {\mathcal {G}}_H(\mathbf {S})^T \right)$ and $\ker \left( \mathbf {S}^T \right)$ have the same dimension and thus are equal. $\square$

#### Corollary 1

Consider a CRN having stoichiometric matrix $\mathbf {S}$, and satisfying the global stability condition of Definition 5, and consider $\mathbf {x}, \ \mathbf {y} \in {\mathbb {R}}_+^n$ such that ${\mathcal {S}}{\mathcal {C}}(\mathbf {x}) = {\mathcal {S}}{\mathcal {C}}(\mathbf {y})$. Given $H \subseteq \left\{ 1, \dots , r \right\}$ such that Eq. (12) holds, consider the CRN having stoichiometric matrix ${\mathcal {G}}_H(\mathbf {S})$, i.e. described by the system of ODEs

14 $$\begin{aligned} \dot{\mathbf {x}}= {\mathcal {G}}_H(\mathbf {S}) \, \mathbf {v}(\mathbf {x},\mathbf {k}) . \end{aligned}$$

If also this projected CRN satisfies the global stability condition, then the trajectories obtained by solving the system of ODEs (14) with initial points $\mathbf {x}$ and $\mathbf {y}$ tend asymptotically to the same steady state.

#### Proof

According to Theorem 2, under the assumption of the Corollary, $\mathbf {S}$ and ${\mathcal {G}}_H(\mathbf {S})$ define the same SCCs. Thus, if $\mathbf {x}$ and $\mathbf {y}$ belong to the same SCC in the original CRN with stoichiometric matrix $\mathbf {S}$, they also belong to the same SCC in the modified CRN with stoichiometric matrix ${\mathcal {G}}_H(\mathbf {S})$. As a consequence, the thesis follows from Definition 5, since the CRN having stoichiometric matrix ${\mathcal {G}}_H(\mathbf {S})$ satisfies the global stability condition and thus for every SCC there exists a unique global asymptotically stable state. $\square$

#### Remark 8

This holds in particular if $\mathbf {x}$ and $\mathbf {y}$ are replaced by the initial state $\mathbf {x}_0$ and the corresponding steady state $\mathbf {x}_e$, respectively. When considering the CRN identified by ${\mathcal {G}}_H(\mathbf {S})$ the trajectories starting from $\mathbf {x}_0$ and $\mathbf {x}_e$ lead to the same mutated steady state.

### Concatenation of mutation

Many cancers arise by effect of a series of mutations accumulated in the cell over time. Here we generalize the results discussed in the previous sections to model the simultaneous action of multiple mutations on a given cell.

For the sake of clarity, we first provide a simple example on how multiple mutations can be incorporated in the network showing that the resulting steady state does not depend on the order of such mutations. The general result shall be proved in Theorem 3. Consider for example a cell affected by two mutations resulting in the LoF of the elemental species $A_{j_1}$ and $A_{j_2}$. The most natural approach to quantify the combined effect of the two mutations is to start from a concentration vector $\mathbf {x}_e$ modeling the (steady) state of a cell in physiological condition, and then apply the procedure described in Sect. 3.1 for each single mutation one after the other. Specifically, we first model the effect of the LoF of $A_{j_1}$ by projecting $\mathbf {x}_e$ through ${\mathcal {P}}_{L_{j_1}}$ and by computing the asymptotically stable state of the system of ODEs (1) with initial condition

15 $$\begin{aligned} \mathbf {x}(0) = {\mathcal {P}}_{L_{j_1}}(\mathbf {x}_e) . \end{aligned}$$

Let $\mathbf {x}_e^{(j_1)}$ be the resulting mutated steady state. As observed in Remark 5, both ${\mathcal {P}}_{L_{j_1}}(\mathbf {x}_e)$ and $\mathbf {x}_e^{(j_1)}$ belong to the SCC

16 $$\begin{aligned} \left\{ \mathbf {y} \in {\mathbb {R}}_+^n \text { s.t. } \mathbf {N}\mathbf {y} = \mathbf {c}^{(j_1)} \right\} , \end{aligned}$$

where $c_i^{(j_1)} = c_i \, (1-\delta _{ij_1})$, $i = 1, \dots , p$, and $\mathbf {c} := \mathbf {N} \mathbf {x}_e$. Then we use ${\mathcal {P}}_{L_{j_2}}$ to project $\mathbf {x}_e^{(j_1)}$, and we compute the solution of the system of ODEs (1) with initial condition

17 $$\begin{aligned} \mathbf {x}(0) = {\mathcal {P}}_{L_{j_2}}(\mathbf {x}^{(j_1)}_e) . \end{aligned}$$

The solution of this Cauchy problem and the corresponding asymptotically stable state $\mathbf {x}_e^{(j_1j_2)}$ will belong to the SCC

18 $$\begin{aligned} \left\{ \mathbf {y} \in {\mathbb {R}}_+^n \text { s.t. } \mathbf {N}\mathbf {y} = \mathbf {c}^{(j_1j_2)} \right\} \end{aligned}$$

where $$c_i^{(j_1j_2)} = c_i \, (1-\delta _{ij_1})(1-\delta _{ij_2})$$, $$i = 1, \dots , p$$.

If the CRN satisfies the global stability condition, the same final steady state $\mathbf {x}_e^{(j_1j_2)}$ may be obtained by reversing the order of the mutations. First we project $\mathbf {x}_e$ through ${\mathcal {P}}_{L_{j_2}}$ and we compute the steady state $\mathbf {x}_e^{(j_2)}$ of the system of ODEs (1) with initial condition

19 $$\begin{aligned} \mathbf {x}(0) = {\mathcal {P}}_{L_{j_2}}(\mathbf {x}_e) . \end{aligned}$$

Then we apply ${\mathcal {P}}_{L{j_1}}$ to $\mathbf {x}_e^{(j_2)}$ and we consider the system of ODEs (1) with initial condition

20 $$\begin{aligned} \mathbf {x}(0) = {\mathcal {P}}_{L_{j_1}}(\mathbf {x}^{(j_2)}_e) . \end{aligned}$$

From Remark 5 it follows that the resulting solution will belong to the SCC defined in (16) and thus it tends asymptotically to the unique stable state $\mathbf {x}_e^{(j_1j_2)}$, because the CRN satisfies the global stability condition.

Additionally, the steady state $\mathbf {x}_e^{(j_1j_2)}$ can be computed in a single step by solving the ODEs system (1), with initial value the concentration vector ${\mathcal {P}}_{L_{j_2}} \circ {\mathcal {P}}_{L_{j_1}}(\mathbf {x}_e)$ that lies on the SCC (16). As we shall prove in Theorem 3, this result easily generalizes to an arbitrary set of combined GoF and LoF mutations through the following definition.

#### Definition 8

Consider an elemented CRN described by the system (1) with stoichiometric matrix $\mathbf {S}$. Consider a set of $\ell$ mutations, resulting in the LoF of the elemental species $A_{j_1}, \dots , A_{j_\ell }$, and a set of *q* GoF mutations, resulting in the suppression of the sets of reactions $H_1, \dots , H_q \subseteq \left\{ 1, \dots , r \right\}$.

The combined effect of the considered mutations is quantified by computing the asymptotically stable state of the modified system of ODEs $\dot{\mathbf {x}}= \tilde{\mathbf {S}} \, \mathbf {v}(\mathbf {x},\mathbf {k})~$, with stoichiometric matrix

21 $$\begin{aligned} \tilde{\mathbf {S}} =\ {\mathcal {G}}_{H_{q}} \circ \dots \circ {\mathcal {G}}_{H_1}(\mathbf {S}) \end{aligned}$$

and initial condition

22 $$\begin{aligned} \mathbf {x}(0) = {\mathcal {P}}_{L_{j_{\ell }}} \circ \dots \circ {\mathcal {P}}_{L_{j_1}}(\mathbf {x}_e) \end{aligned}$$

where $\circ$ denotes function composition and $\mathbf {x}_e$ are the values of species concentration reached by the cell in physiological condition.

#### Theorem 3

The steady state computed through the procedure described in Definition 8 does not depend on the order of the composition in the projectors defined in (21) and (22) provided that

23 $$\begin{aligned} \mathrm{rank} \left( {\mathcal {G}}_{H_{q}} \circ \dots \circ {\mathcal {G}}_{H_1}(\mathbf {S}) \right) = \mathrm{rank}(\mathbf {S}) \end{aligned}$$

and that the original CRN as well as the projected system of ODEs satisfy the global stability condition of Definition 5.

#### Proof

The thesis follows by observing that the projectors defined in (21) and (22) do not depend on the order of the composition. Indeed the mutated stoichiometric matrix ${\mathcal {G}}_{H_{q}} \circ \dots \circ {\mathcal {G}}_{H_1}(\mathbf {S})$ is defined by setting to zero the columns of $\mathbf {S}$ corresponding to the reactions in $H_1 \cup \dots \cup H_q$, and hence it does not depend on the order in which the GoF mutations are considered. Additionally if condition (23) holds then ${\mathcal {G}}_{H_{q}} \circ \dots \circ {\mathcal {G}}_{H_1}(\mathbf {S})$ and $\mathbf {S}$ define the same SCCs as shown in Theorem 2. The initial condition (22) defines the unique SCC

$$\begin{aligned} {\mathcal {S}}{\mathcal {C}}({\mathcal {P}}_{L_{j_{\ell }}} \circ \dots \circ {\mathcal {P}}_{L_{j_1}}(\mathbf {x}_e)) = \left\{ \mathbf {x}\in {\mathbb {R}}^n_+ \text { s.t. } \mathbf {N}\mathbf {x} = \widetilde{\mathbf {c}} \right\} \end{aligned}$$

where $\widetilde{\mathbf {c}}$ has been obtained from $\mathbf {c} := \mathbf {N}\mathbf {x}_e$ by setting to zero the $j_i-$th element, for all $i = 1, \dots \ell$. A different order of the LoF mutations results in a different initial concentration vector on the same SCC. Therefore, according to Theorem 1 it leads to the same mutated asymptotically steady state. $\square$

#### Remark 9

As proved in Theorem 3, the order in which mutations are considered in our model does not impact the final steady state reached by the mutated network. This is a crucial result coherent with the fact that our CRC–CRN models the G1-S transition of a single colorectal cell. Future effort would be dedicated to extend our model to account for e.g. the selection process induced on the mutated cell by the external environment, process that may explain also more recent experimental studies showing the importance of the order of mutations on the progression of cancer diseases (Levine et al. 2019).

#### Remark 10

The procedure described by Definition 8 provides an efficient approach to simultaneously quantify the effects of a group of mutations. However, if one is interested in differentiating the effects of each mutation on the whole process leading from $\mathbf {x}_e$ to the final mutated equilibrium, the procedure in Definition 8 is equivalent to the following iterative procedure. After accounting for the first GoF mutation by computing the asymptotically stable state $\mathbf {x}_e^{(H_1)}$ of the projected Cauchy problem

24 $$\begin{aligned} {\left\{ \begin{array}{ll} \dot{\mathbf {x}}= {\mathcal {G}}_{H_1}(\mathbf {S}) \, \mathbf {v}(\mathbf {x},\mathbf {k})\\ \mathbf {x}(0) = \mathbf {x}_e \end{array}\right. } , \end{aligned}$$

we quantify the effect of the *i*-th GoF mutation, $i = 2, \dots , q$, by computing the asymptotically stable state $\mathbf {x}_e^{(H_1 \dots H_i)}$ of the projected Cauchy problem

25 $$\begin{aligned} {\left\{ \begin{array}{ll} \dot{\mathbf {x}}= {\mathcal {G}}_{H_{i}} \circ \dots \circ {\mathcal {G}}_{H_1}(\mathbf {S}) \, \mathbf {v}(\mathbf {x},\mathbf {k})\\ \mathbf {x}(0) = \mathbf {x}_e^{(H_1 \dots H_{i-1})} \end{array}\right. } . \end{aligned}$$

We then proceed with the LoF mutations. First we compute the asymptotically stable state $\mathbf {x}_e^{(H_1 \dots H_q j_1)}$ of the Cauchy problem

26 $$\begin{aligned} {\left\{ \begin{array}{ll} \dot{\mathbf {x}}= {\mathcal {G}}_{H_{q}} \circ \dots \circ {\mathcal {G}}_{H_1}(\mathbf {S}) \, \mathbf {v}(\mathbf {x},\mathbf {k})\\ \mathbf {x}(0) = {\mathcal {P}}_{L_{j_1}}(\mathbf {x}_e^{(H_1 \dots H_{q})}) \end{array}\right. } . \end{aligned}$$

Then for each $i = 2, \dots , \ell$, we account for the *i*-th LoF mutation by computing the asymptotically stable state $\mathbf {x}_e^{(H_1 \dots H_{q}j_1 \dots j_{i})}$ of the Cauchy problem.

27 $$\begin{aligned} {\left\{ \begin{array}{ll} \dot{\mathbf {x}}= {\mathcal {G}}_{H_{q}} \circ \dots \circ {\mathcal {G}}_{H_1}(\mathbf {S}) \, \mathbf {v}(\mathbf {x},\mathbf {k})\\ \mathbf {x}(0) = {\mathcal {P}}_{L_{j_{i}}} \circ \dots \circ {\mathcal {P}}_{L_{j_1}}(\mathbf {x}_e^{(H_1 \dots H_{q}j_1 \dots j_{i-1})}) \end{array}\right. } . \end{aligned}$$

If Eq. (23) holds and all the projected systems of ODEs defined in (24)–(27) satisfy the global stability condition, the final equilibrium point $\mathbf {x}_e^{(H_1 \dots H_{q}j_1 \dots j_{\ell })}$ is equal to the stable state obtained with the procedure described in Definition 8. This can be easily proved by defining $\mathbf {c} := \mathbf {N}\mathbf {x}_e$ and observing that, under the previous assumptions, $\mathbf {x}_e^{(H_1)} \in \left\{ \mathbf {x} \in {\mathbb {R}}^n_+ \text { s.t. } \mathbf {N}\mathbf {x} = \mathbf {c} \right\}$, $\mathbf {x}_e^{(H_1H_2)} \in \left\{ \mathbf {x} \in {\mathbb {R}}^n_+ \text { s.t. } \mathbf {N}\mathbf {x} = \mathbf {N}\mathbf {x}_e^{(H_1)} = \mathbf {c} \right\}$ and thus inductively $\mathbf {x}_e^{(H_1H_2 \dots H_q)} \in \left\{ \mathbf {x} \in {\mathbb {R}}^n_+ \text { s.t. } \mathbf {N}\mathbf {x} = \mathbf {N}\mathbf {x}_e^{(H_1 \dots H_{q-1})} = \mathbf {c} \right\}$. Analogously it can be inductively proved that $\mathbf {x}_e^{(H_1H_2 \dots H_qj_1 \dots j_{\ell })}$ belongs to the SCC ${\mathcal {S}}{\mathcal {C}}({\mathcal {P}}_{L_{j_{\ell }}} \circ \dots \circ {\mathcal {P}}_{L_{j_1}}(\mathbf {x}_e))$ defined in the proof of Theorem 3. Thus the thesis follows from the fact that all the projected ODEs systems satisfy the global stability condition.

Obviously, changing the order of the mutations affects the trajectory covered by the described iterative procedure but will lead to the same final steady state.

## Application to the colorectal cancer cells

### Generalities

In this section we apply the previous procedures to the analysis of a CRN which has been devised to provide a simplified description of how signals carried by a ligand from outside the cell are processed in order to determine the behavior of a possibly cancerous colorectal cell. The starting point of our analysis is the kinetic model originally proposed by Tortolina et al. (2015), to model a healthy cell entering the G1-S phase of its development, when a synthesis of new DNA starts, as a first step in the process of cell division. Following the analysis of the previous sections, in this work the model is adapted to formally describe mutations that transform healthy cells into cancer ones; two typical cases of LoF and GoF mutations are then numerically simulated, and the resulting mutated equilibrium concentrations are compared with the corresponding healthy values.

Henceforth, we shall refer to this signaling network for healthy and CRC cells as CRC–CRN. The basic idea underlying the CRC–CRN approach developed in Tortolina et al. (2015) is that CRC may be associated with mutations of signaling-proteins. An effective insight into the role of each mutation can only be obtained if mutated and healthy proteins are integrated into pathways, which in turn are incorporated into a comprehensive network. For example, this is a necessary step in order to perform numerical simulations of the effects induced by possible targeted drugs. As already observed, the CRC–CRN is focused on the G1-S transition phase of the cell cycle; moreover, it results from the interconnection of pathways activated by external signals through the TGF$\beta$, WNT, and EGF families of receptor ligands. The protein species, as well as the chemical reactions inserted into the network have been chosen on the basis of literature data; this holds also for the values of rate constants. Tortolina et al. (2015) validated the predictive power of the CRC–CRN by comparing the results provided by their model with more recent published data, independent of those used to formulate and train the original model. Additionally, the response of mutated HCT116 and HT29 CRC lines to perturbing inhibitors has been experimentally found to agree with the predictions of the model.

The most relevant features of the CRC–CRN are summarized below; the full list of chemical species together with their abbreviated names, the set of chemical reactions, the system of ODEs, the fixed values of the rate constants, and the initial concentrations of the elemental variables in an ideal physiological state can be found in the Supplementary Materials of Tortolina et al. (2015). The CRC–CRN involves 8 constant, and 411 varying chemical species. The constant species model non-consumable chemicals such as the three growth factors TGF$\beta$, WNT, and EGF. Internal interactions are modeled by 339 reversible and 172 irreversible reactions, for a total of $r = 850$ reactions. Chemical kinetics is based on mass action law, so that 850 rate constants are considered. The state of CRC–CRN is described by $n = 419$ variables resulting from the concentrations of the 411 internal species, plus 8 additional variables, of null time derivative, accounting for the constant inflows. The state variables satisfy a system of 419 polynomial ODEs as in Eq. (1). In particular the monomials defining the reaction fluxes ${\mathbf {v}}({\mathbf {x}}, {\mathbf {k}})$ are quadratic, since the reactants considered depend at most on two chemical species. The rate constants enter the system as real and positive parameters.

### Conservation laws and elemental variables of the CRC–CRN

We have obtained a basis of $\ker (\mathbf {S}^T)$ consisting of $p=81$ semi-positive conservation vectors, providing an independent set of 81 CLs, all of them being regarded as moiety CLs. In particular, we point out that CRC–CRN satisfies the condition $p = n - \mathrm{rank}\, (\mathbf {S})$. As expected, 8 CLs correspond to constancy of original non-consumable chemical species, so that they can be considered as trivial. The remaining 73 CLs describe effective properties of the system.

It is seen by inspection that the CRC–CRN is weakly elemented, with 9 chemical species not involved in the conservation laws. Up to the 8 constant species, the list of elemental species coincides with that of the basic species of Tortolina et al. (2015), which were defined as consisting of proteins in free form, which may bind to other species in order to form derived compounds or secondary species.

A simple, rather typical, example of conservation law within our CRC–CRN is

28 $$\begin{aligned} \text {NLK} + \text {NLKP} + \text {NLKP}_-\text {TCFLEF}+ \text {NLKP}_-\text {Pase10} + \text {TAKP}_-\text {TAB}_-\text {NLK}= c \end{aligned}$$

where *c* is a given constant; here NLK denotes the concentration of the elemental species “Nemo like kinase” (Tortolina et al. 2015); NLKP is the phosphorylated form of NLK, while an expression like NLKP$_-$TCFLEF refers to the compound formed by NLKP and TCFLEF. Equation (28) expresses conservation in time of the total number of molecules of NLK belonging to the compounds entering the combination in the left side, as discussed in Sect. 2.1. In words, the NLK molecules are transferred between the metabolites involved in (28), but are not synthesised, degraded or exchanged with the environment (De Martino et al. 2014; Haraldsdóttir and Fleming 2016). The constant *c*, which is determined by the initial conditions, may be regarded as a counter of the conserved molecules of NLK.

### Global stability of CRC–CRN

There are a number of results available for equilibrium points and their stability properties (Chellaboina et al. 2009; Domijan and Kirkilionis 2008; Feinberg 1995; Yu and Craciun 2018; Conradi and Flockerzi 2012; Conradi and Mincheva 2014), but they cannot be applied straightforwardly to this CRC–CRN, essentially for two reasons. In fact, some of these results require very technical hypotheses that cannot be verified in the case of our ODEs system, due to its dimension and complexity. Further, other results cannot be applied in the case of open systems, like the one considered in this paper. Therefore, we will make use of numerical simulations to support the following conjecture.

#### Conjecture 1

The CRC–CRN described by system (1), with stoichiometric matrix ${\mathbf {S}}$ and rate constants $\mathbf{k }$ set as proposed by Tortolina et al. (2015), satisfies the global stability condition introduced in Definition 5.

*Numerical verification* We defined 5 different SCCs by randomly selecting the values of the total concentrations $\mathbf {c}$. Specifically, each element $c_j$, $j = 1, \dots , p$, has been drawn from a $\log _{10}$-uniform distribution on $[10^{-2}, 10^3]$. For each of the obtained SCC we generated 30 initial points

$$\begin{aligned} \mathbf {x}^{(k)}_0 \in \left\{ \mathbf {y} \in {\mathbb {R}}^n_+\ \text {s.t.}\ \mathbf {N}\mathbf {y} = \mathbf {c} \right\} , \ k = 1, \dots , 30\, \end{aligned}$$

as follows.

- First, we dealt with the species that do not belong to any conservation law. The value of their initial concentration were $\log _{10}$-uniformly drawn from the interval $[10^{-5}, 10^5]$.
- Then we considered all the other chemical species but the elemental species. After randomly permuting their order, for each species *i*:
  1. we randomly selected an initial concentration value below the upperbound imposed by the total concentrations available in the conservation laws involving it. More in details we set
    $$\begin{aligned} x^{(k)}_{0, i} = u \cdot \min _{j \in \Gamma (i)}{\frac{c_j}{\gamma _{ji}}} , \end{aligned}$$
    where *u* was uniformly drawn from [0, 1] and
    $$\begin{aligned} \Gamma (i) = \left\{ j \in \{1, \dots , p\}\ \text {s.t.}\ \gamma _{ji} \ne 0 \right\} . \end{aligned}$$
    is the set of all the conservation laws involving the $i-$th species.
  2. we updated the total concentration available in each conservation law by removing the amount already filled by the $i-$th species, i.e.
    $$\begin{aligned} c_j \leftarrow c_j - \gamma _{ji} \ x^{(k)}_{0, i} \end{aligned}$$
    for all $$j \in \Gamma (i)$$.
- Finally we considered the elemental species. By exploiting the fact that each elemental species belongs to only one conservation law, *j*, we set
  $$\begin{aligned} x^{(k)}_{0, i} = c_j \end{aligned}$$
  where $c_j$ is the value of the total concentration still available after the previous step.

For each of the 30 points generated with the described procedure, we used the matlab tool ode15s (Shampine and Reichelt 1997) to integrate the system of ODEs (1) on the interval $[0, 2.5 \cdot 10^7]$, with initial condition $\mathbf {x}(0) = \mathbf {x}_0^{(k)}$. The value of the solution at the last time-point was considered as the corresponding asymptotic steady state $\mathbf {x}_e^{(k)}$.

Since the initial values $\mathbf {x}_0^{(k)}$ all belong to the same SCC they should lead to the same steady state. This was verified by computing for each species the coefficient of variation of the equilibrium values across the 30 trajectories. Namely, for each species we computed

29 $$\begin{aligned} \varepsilon _e(i) = \frac{\sqrt{\frac{1}{29} \sum _{k=1}^{30} ( x_{e, i}^{(k)} - \mu _e(i) )^2 }}{\mu _e(i)} \end{aligned}$$

where $$\mu _e(i) = \frac{1}{30}\sum _{k=1}^{30} x_{e, i}^{(k)}$$.

As a comparison, for each species we also computed the coefficient of variation $\varepsilon _0(i)$ across the initial values $x_{0, i}^{(k)}$, $k = 1, \dots , 30$.

**Fig. 1:**
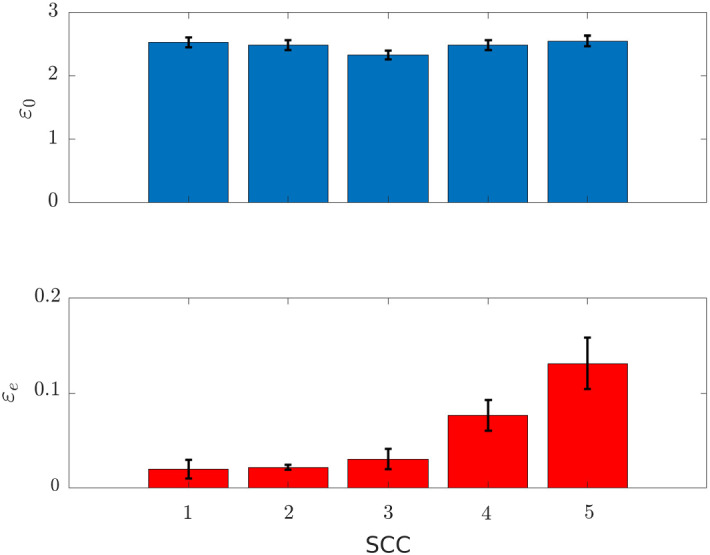
Average and standard error of the mean over the non–constant species of the coefficient of variation across 30 different initial conditions (upper panel) and the corresponding asymptotically stable states (lower panel). Each bar corresponds to the results obtained with a different SCC. Note the different scale on the *y* axes

Figure 1 shows the averaged coefficient of variations obtained with the 5 considered SCCs. Fixed a SCC, the coefficient of variation across the initial values $\varepsilon _0$ is always around 2.5, while the coefficient of variation across the steady states $\varepsilon _e$ is at least one order of magnitude lower. Moreover, the latter shows higher differences across the SCCs. This is mainly due to the fact that depending on the SCC, few species may require a longer time to reach the asymptotically stable state and thus may show a higher variation when the system of ODEs is integrated in the fixed time-interval $[0, 2.5 \cdot 10^7]$.

### LoF mutation of TGF$\beta$RII

Through a signaling pathway, called the transforming growth factor-$\beta$ (TGF-$\beta$) pathway, the environment outside the cell stimulates activation of genes which control cell growth and division. During early progression of CRC, the TGF-$\beta$ pathway serves as a tumor suppressor, by inhibiting proliferation and accelerating apoptosis; however, LoF of the proteins of the TGF-$\beta$ pathway may weaken the tumor suppressor function, and favor cancer progression. Indeed, it is found in many colon cancer tumors that the binding between the signal protein TGF-$\beta$ and one of its receptors, TGF$\beta$RII, is altered by a LoF mutation of the receptor (Armaghany et al. 2012; Yang and Yang 2010; Zhang 2018). Here we use the CRC–CRN in order to simulate a few consequences of the LoF mutation of the elemental species TGF$\beta$RII.

First, we computed the value of the concentration vector modeling the physiological steady state of the cell prior to mitosis. To this end, we integrated the system of ODEs (1) on the interval $[0, 2.5 \cdot 10^7]$, with initial condition $\mathbf {x}(0) = \mathbf {x}_0$, where $\mathbf {x}_0$ is the ideal state defined by setting the initial concentrations of the elemental species as in Tortolina et al. (2015). The value of the steady state in the physiological cell was defined as the value of the solution at the last time–point.

By using Definition 6, we then defined the operator ${\mathcal {P}}_{L_j}$ associated to the LoF of TGF$\beta$RII. The steady state of the mutated cell was computed by integrating the system of ODEs (1) with two different initial conditions, namely $\mathbf {x}(0) = {\mathcal {P}}_{L_j}(\mathbf {x}_0)$ and $\mathbf {x}(0) = {\mathcal {P}}_{L_j}(\mathbf {x}_e)$. As in the previous step, in both cases we solved the system on the interval $[0, 2.5 \cdot 10^7]$ and we defined as steady state the value of the solution computed at the last time–point.

According to Theorem 1 the two trajectories, starting from ${\mathcal {P}}_{L_j}(\mathbf {x}_0)$ and ${\mathcal {P}}_{L_j}(\mathbf {x}_e)$, should lead to the same steady state. This result was numerically verified by computing for both the trajectories the relative difference

30 $$\begin{aligned} d_i = \frac{x^m_{e, i} - x_{e, i}}{x_{e, i}} , \quad i = 1, \dots ,n \end{aligned}$$

where $\mathbf {x}_e^m$ is the steady state in the mutated cell.

**Fig. 2:**
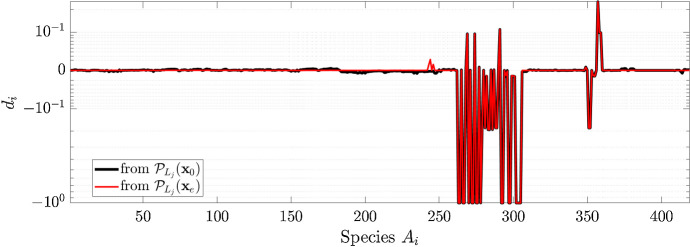
Value of the relative difference $d_i$ between the steady states in the physiological cell and in the cell affected by LoF mutation of TGF$\beta$RII. Black and red lines are obtained when the system of ODEs for the mutated cell is solved with initial condition $\mathbf {x}(0) = {\mathcal {P}}_{L_j}(\mathbf {x}_0)$ and $\mathbf {x}(0) = {\mathcal {P}}_{L_j}(\mathbf {x}_e)$, respectively

As shown in Fig. 2, the results obtained with the two different initial conditions coincide and thus Theorem 1 is verified. Moreover, Fig. 2 shows the effect of the LoF of TGF$\beta$RII on the concentrations of all the chemical species. Indeed, $d_i < 0$ means that the value of the concentration of the $i-$th species in the mutated cell is lower than in the healthy cell. On the contrary $d_i > 0$ means that the amount of the $i-$th species is higher in the mutated cell. A further inspection reveals that few proteins, belonging to SMAD4 pathway, reached a value of $d_i$ equal to $-\,1$, meaning that their function is completely stopped as a consequence of the LoF of TGF$\beta$RII. This is confirmed by different literature data showing a down-regulation of SMAD4 pathway due to alterations in TGF$\beta$RII signaling (Bellam and Pasche 2010; Zhao et al. 2018).

**Fig. 3:**
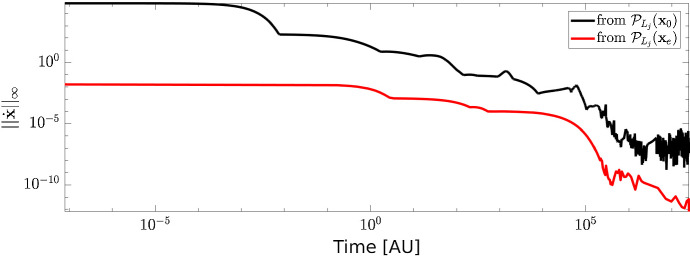
Infinity norm of the derivative $\dot{\mathbf {x}}$ of the concentration vector as function of time. Black and red lines show the results obtained by solving the ODEs system (1) with initial condition $\mathbf {x}(0) = {\mathcal {P}}_{L_j}(\mathbf {x}_0)$ and $\mathbf {x}(0) = {\mathcal {P}}_{L_j}(\mathbf {x}_e)$, respectively, ${\mathcal {P}}_{L_j}$ being the operator associate to the LoF mutation of TGF$\beta$RII

On the other hand, different values of the initial conditions lead to different trajectories. In Fig. 3 we compared the time required by the two trajectories to reach a stable state. To this end for each time point *t* we computed

31 $$\begin{aligned} ||\dot{\mathbf {x}}(t) ||_{\infty } = ||\mathbf {S} \mathbf {v}(\mathbf {x}(t), \mathbf {k}) ||_{\infty } . \end{aligned}$$

Since $\mathbf {x}_e$ is already a steady state for the CRC–CRN, when computing the projected value ${\mathcal {P}}_{L_j}(\mathbf {x}_e)$ the species not affected by the LoF of TGF$\beta$RII maintain their stable values. As a consequence, the trajectory starting from ${\mathcal {P}}_{L_j}(\mathbf {x}_e)$ requires a lower number of iterations to reach a value of $||\dot{\mathbf {x}}(t) ||_{\infty }$ closer to 0.

### GoF mutation of BRAF

The BRAF protein belongs to the mitogen-activated protein kinase (MAPK) pathway, which represents a major cell proliferation signal transduction pathway from the cell surface to the nucleus. The MAPK pathway, following activation by the epidermal growth factor (EGF) protein, regulates the growth and division (proliferation) of cells, the process by which cells mature to carry out specific functions (differentiation), cell movement (migration), and the self-destruction of cells (apoptosis). In a normal cell, the BRAF protein is switched on and off in response to signals that control cell growth and development. In consequence of a mutation, a BRAF protein may be continuously active, transmitting messages to the nucleus even in the absence of these chemical signals. Thus, the overactive BRAF protein may contribute to the growth of cancers by allowing abnormal cells to grow and divide without the mediation of external signals (Armaghany et al. 2012; Morkel et al. 2015).

We investigated the impact on the CRC–CNR of a mutation resulting in the GoF of the elemental species BRAF.

To this end, following Definition 7, we defined the operator ${\mathcal {G}}_H$, where the *H* is defined to include all the reactions involved in the deactivation of BRAF$^*$. We recall that BRAF$^*$ is the activated form of BRAF, consisting in the phosphorylation of a specific amino acid. Thus it is assumed that the mutated form of BRAF is still subject to activation in BRAF$^*$, while inactivation of BRAF$^*$ is blocked.

As described in Tortolina et al. (2015), Supplementary materials, the deactivation of BRAF$^*$ is regulated by the phosphatase Pase1 through the following set of reactions:

$$\begin{aligned}&\text {BRAF}^{*} + \text {Pase1} \; \overset{k_{1f}}{\underset{k_{1r}}{\rightleftarrows }} \; \text {BRAF}^* _-\text {Pase1}\\&\quad \text {BRAF}^{*} _-\text {Pase1} \; \overset{k_2}{\rightarrow }\; \text {BRAF} + \text {Pase1} \end{aligned}$$

where a simplified notation has been adopted for the rate constants. To block such a deactivation process while respecting the condition described by Eq. (12) in Theorem 2 we removed the two forward reactions

$$\begin{aligned} \ \text {BRAF}^{*} + \text {Pase1} \; \overset{k_{1f}}{\rightarrow }\; \text {BRAF}^{*} _-\text {Pase1} \; \overset{k_2}{\rightarrow }\; \text {BRAF} + \text {Pase1} , \end{aligned}$$

that is we defined a novel stoichiometric matrix ${\mathcal {G}}_H(\mathbf {S})$ were the corresponding columns of $\mathbf {S}$ have been set equal to 0.

Similarly to what we have done in the previous section, we computed the asymptotically stable states of the trajectories obtained solving the system of ODEs defined by the stoichiometric matrix ${\mathcal {G}}_H(\mathbf {S})$ with two different initial condition, namely $\mathbf {x}(0) = \mathbf {x}_0$ and $\mathbf {x}(0) = \mathbf {x}_e$, $\mathbf {x}_0$ and $\mathbf {x}_e$ being the ideal and steady state for the physiological cell.

**Fig. 4:**
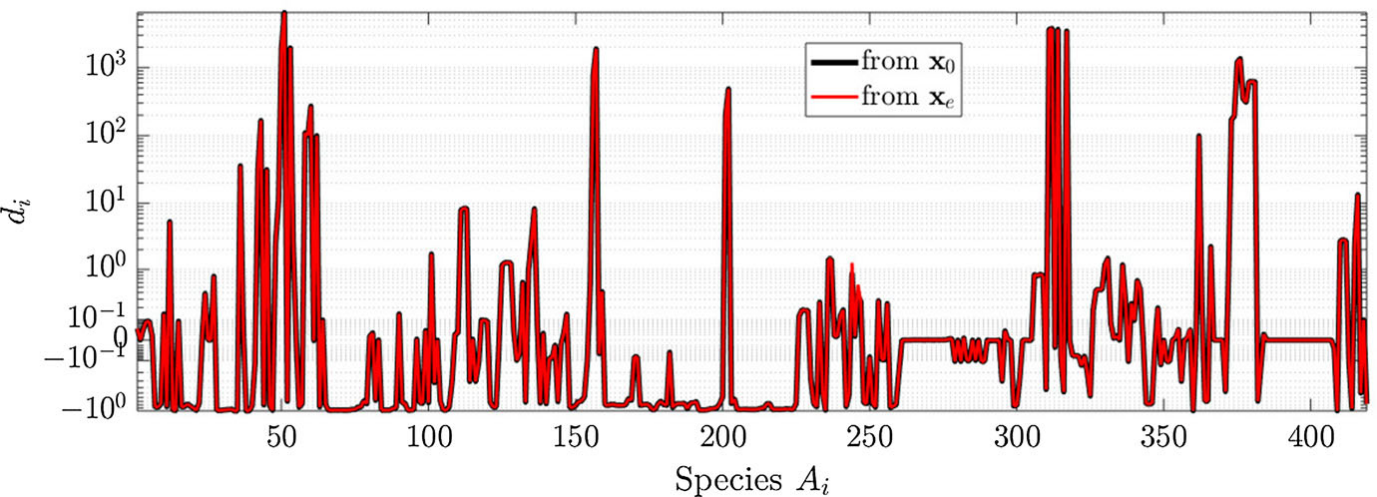
Value of the relative difference $d_i$ between the steady states in the physiological cell and in the cell affected by GoF mutation of BRAF. Black and red lines are obtained when the mutated system of ODEs defined by the stoichiometric matrix ${\mathcal {G}}_H(\mathbf {S})$ is solved with initial condition $\mathbf {x}(0) = \mathbf {x}_0$ and $\mathbf {x}(0) = \mathbf {x}_e$, respectively

**Fig. 5:**
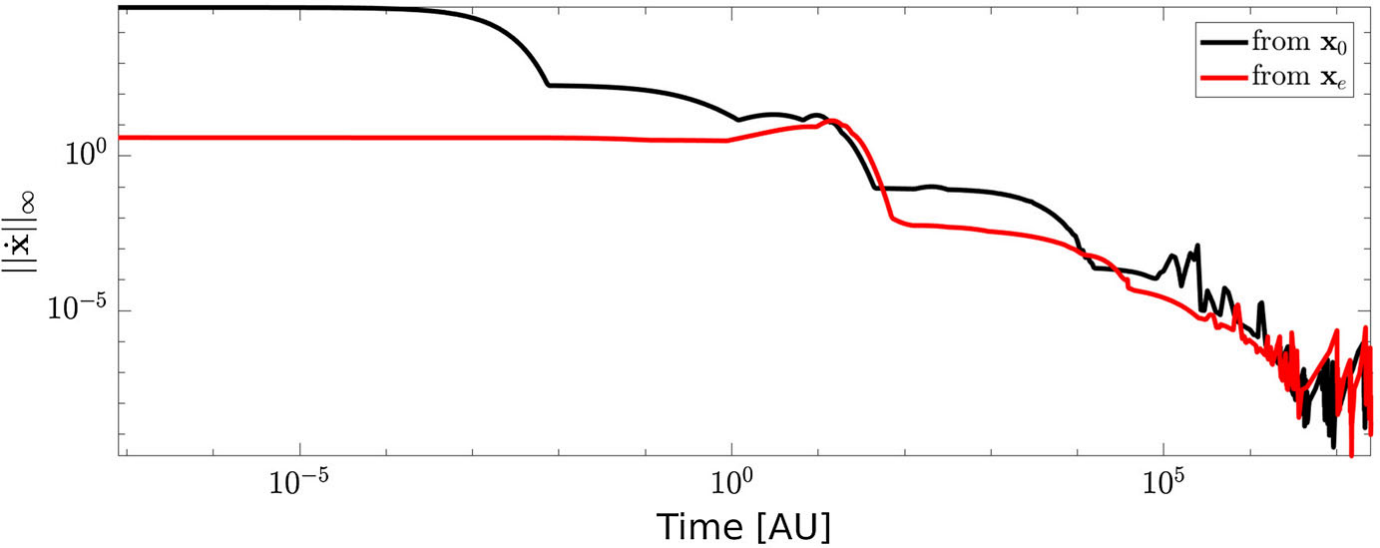
Infinity norm of the derivative $\dot{\mathbf {x}}$ of the concentration vector as function of time when the stoichiometric associated to GoF of BRAF is employed in the ODEs system (1). Black and red lines show the results obtained by setting $\mathbf {x}(0) = \mathbf {x}_0$ and $\mathbf {x}(0) = \mathbf {x}_e$, respectively

Figure 4 shows that the two trajectories lead to the same steady state as stated in Corollary 1. Additionally, as done in the previous section, in Fig. 5 we compare the time required by the two trajectories to reach the stable state. According to Definition 7, the GoF of BRAF is implemented by setting to zero a set of columns of the stoichiometric matrix ${\mathbf {S}}$, and thus modifying the system of ODEs associated to the CRC–CRN. Although $\mathbf {x}_e$ was a stable state for the system of ODEs modeling the cell in physiological condition, for most of the species the corresponding concentration $x_{e, i}$ is no longer an equilibrium value for the new system. For this reason the two trajectories, starting from $\mathbf {x}_e$ and $\mathbf {x}_0$, show similar behaviors of $||\dot{\mathbf {x}}(t) ||_{\infty }$ as function of the time *t* in which the solution of the system is computed.

As a final remark, we observe that, as shown in Fig. 4, the concentrations of a high number of proteins are altered by the GoF of BRAF, and in general the values of $d_i$ are bigger than those found with the LoF of TGF$\beta$RII, see Fig. 2. These results highlight the central role within the CRC–CRN of BRAF, that in fact is the target of various therapies (Morkel et al. 2015).

## Discussion and future directions

In this paper we have considered a system of ODEs modeling a CRN, with emphasis on the mutual relationships between moiety CLs and stoichiometric surfaces. CLs have led to the characterization of weakly elemented CRN and the definition of ideal states, which are crucial in the treatment of mutations. Confining attention to systems satisfying the global stability condition, we have considered two classes of mutations, LoF and GoF, often found in cancer cells. Mutations have been modeled as projection operators and basic properties of mutated networks have been analyzed, aiming at the determination of mutated equilibrium states as asymptotic steady limits of trajectories. The new model for mutations allows for a simple treatment of their combinations Furthermore, if the original and the projected CRNs satisfy the global stability condition, the resulting equilibrium is independent of the order in which mutations are combined.

As an application of the previous results we have investigated a system of ODEs describing the G1-S phase of a CRC cell. Due to the huge number of variables involved, the analysis has been based on numerical simulations. We have found 81 independent, linear, moiety CLs; they have been applied to support the conjecture that CRC–CRN satisfies the global stability condition. It has also been found that the elemental chemical species coincide with the basic species defined by Tortolina et al. (2015) on biochemical grounds. Also, a mutation by LoF, and another mutation by GoF have been examined in detail. Specifically, our numerical simulations highlighted the relationship between the LoF of TGF$\beta$RII and the down-regulation of SMAD4 pathway, and confirmed the central role of protein BRAF within colorectal cell signaling. A more thorough discussion of the possible biological applications of the proposed model will be the subject of a second study.

We are aware that this computational approach to CRN is devised to capture few, although decisive, aspects of the G1-S transition point of a cell, as well as the modifications of the network induced by cancer mutations. We think that the model can provide a guide to future experimental research based on the interpretation of results of simulations, particularly in the case of the development and optimization of targeted therapies against already mutated cells. For example, if the simulation predicts a significant increase of the mutated concentration of a specific metabolite with respect to its physiological value, then that metabolite can be regarded as a potential drug target. More in general, an analysis of the mutated profile of the simulated CRN in the G1-S phase should provide hints about convenient choices of targets for available drugs, together with a framework for the simulation of their consequences (Facchetti et al. 2012; Torres and Altafini 2016).

Possible development of our model may involve different directions. For example, we could extend it to examine alterations of mRNA induced by changes from physiological to mutated equilibrium (Castagnino et al. 2016). Other classes of mutation may be considered. For example, a mutation resulting in the reduction of the catalyzing activity of an enzyme may be modeled by setting to zero the rate constant of the corresponding reactions, a strategy similar to the one we used for the GoF mutations. Moreover, a mutation resulting in a continuous activation of a given transcription factor, or more in general of a given protein *P*, may be modeled by adding to the network a new pseudo-reaction of the form $\emptyset \rightarrow P$, where $\emptyset$ is the so-called zero complex (Feinberg 1987), and thus by enlarging the system (1) with an additional auxiliary variable having null derivative. Further, in the model we have not considered possible dependence of the literature data on temperature, pH, or other conditions. Therefore, the scheme is not capable of describing the individual response of a fixed cell; rather, it attempts at simulating the behavior of a homogeneous set of cells; from this viewpoint, the solution of the system of ODEs may be interpreted as providing an average dynamic of the intra-cellular answer. Also, the scheme should be enriched in order to account for the impact of the extra-cellular micro-environment, which implies to modify the model in order to account for growth-induced solid stresses and pressure, nutrients and oxygen supply, and blood perfusion (Caviglia et al. 2014; Jones and Chapman 2012; Markert and Vazquez 2015).

As a final comment, we remark that the rate constants in the CRC–CRN considered in this paper are assumed as known and estimated from the scientific literature (Tortolina et al. 2015). A more reliable determination of these constants can be obtained by solving a non-linear ill-posed problem (Bertero and Piana 2006; Engl et al. 2009) in which the input data are provided by ad hoc conceived biochemical experiments. The setup of such experiments and the realization of an optimization method for the numerical solution of this inverse problem, together with a corresponding sensitivity and bifurcation analysis, will be part of future research.
